# Podocytes and Proteinuria in ANCA-Associated Glomerulonephritis: A Case-Control Study

**DOI:** 10.3389/fimmu.2019.01405

**Published:** 2019-06-26

**Authors:** Emma E. van Daalen, Peter Neeskens, Malu Zandbergen, Lorraine Harper, Alexandre Karras, Augusto Vaglio, Janak de Zoysa, Jan A. Bruijn, Ingeborg M. Bajema

**Affiliations:** ^1^Department of Pathology, Leiden University Medical Center, Leiden, Netherlands; ^2^School of Immunity and Infection, College of Medical and Dental Sciences, University of Birmingham, Birmingham, United Kingdom; ^3^Nephrology Department, HEGP Hospital, Assistance Publique Hôpitaux de Paris, Paris, France; ^4^Nephrology Unit, Meyer Children's Hospital and Department of Biomedical Experimental and Clinical Sciences “Mario Serio”, University of Firenze, Firenze, Italy; ^5^Renal Services, North Shore Hospital, Auckland, New Zealand

**Keywords:** podocyte, proteinuria, ANCA, vasculitis, renal biopsy

## Abstract

Proteinuria has been identified as prognosticator of renal outcome in patients with ANCA-associated glomerulonephritis, but whether proteinuria is related to podocyte abnormalities in these patients is largely unknown. We here investigate podocyte foot process width and number of podocytes positive for the podocyte marker WT-1 in diagnostic renal biopsies of 25 Caucasian patients with ANCA-associated glomerulonephritis in relation to proteinuria. Control tissue was used from pre-transplantation donor kidney biopsies. Proteinuria at 10 weeks follow-up correlated significantly with foot process width (*P* = 0.04). Biopsies with foot process width ≥600 nm belonged more often to the crescentic or mixed class, whereas biopsies with a foot process width <600 nm were most often categorized as focal class (*P* = 0.03). The mean number of podocytes based upon expression of WT-1 was significantly lower in patients compared to controls (15 vs. 34 podocytes per glomerulus; *P* < 0.0001). The significant decrease in expression of the podocyte WT-1 marker in ANCA-associated glomerulonephritis is considered indicative of actual podocyte loss or at least, of a loss of functionality. Furthermore, our study indicates that podocyte foot process width at baseline could be indicative for proteinuria at short term follow up. For prognostic purposes, we therefore suggest to include a description of the foot process width in the diagnostic report of a biopsy with ANCA-associated glomerulonephritis.

## Introduction

In the patient care and research of anti-neutrophil cytoplasmic antibody (ANCA-) associated glomerulonephritis (AAGN), proteinuria is a subject matter which so far received relatively little attention. Studies on AAGN have mainly focused on renal function deterioration in combination with findings in the urine sediment. However, there are some data indicating that the degree of proteinuria at diagnosis is associated with renal outcome in patients with AAGN (1–3). Also, preliminary data combined from three European Vasculitis Society (EUVAS) clinical trials show that the level of proteinuria during follow-up is a prognostic marker of chronic kidney disease progression (4). At disease presentation, the majority of patients with AAGN have proteinuria, the amount of which is quite variable (5).

In general, the presence of proteinuria in kidney diseases is associated with changes in podocyte morphology (6, 7). Podocytes are highly specialized epithelial cells that, together with the glomerular basement membrane (GBM) and glomerular endothelial cells, constitute the filtration barrier of the glomerular capillary wall. The notion that podocytes react to injury by effacement is generally accepted, but exactly how this reactive change relates to the level of proteinuria, remains a matter of debate (8). Two recent studies investigating foot process effacement in different human glomerulopathies suggested that the amount of foot process effacement is related to the type of glomerulopathy rather than to the amount of proteinuria; for example, patients with IgA nephropathy and minimal change nephrotic syndrome had similar proteinuria levels at diagnosis, but foot processes were significantly more effaced in minimal change nephrotic syndrome (9, 10).

To study podocyte morphology, images at high magnification with electron microscopy (EM) of the podocytes are required. In most centers, EM is not routinely performed in AAGN, because the characteristic findings by light microscopy (LM) and the pauci-immune pattern by immunofluorescence are usually diagnostic. A number of studies investigated EM samples from patients with AAGN (11–15), but only one described the podocyte morphology in detail (16). This was a recent study from China showing that foot process width (FPW) was significantly higher and that podocyte density was significantly lower in an Asian group of patients with AAGN compared to healthy controls. In the current study, we investigate the podocyte morphology and number in renal biopsies of a Caucasian population of patients with AAGN. We analyzed whether and how these parameters were related to proteinuria at baseline and during follow-up.

## Methods

### Study Population

Patients with histopathologically proven AAGN were retrieved from the Pathology database at Leiden University Medical Center, the Netherlands. Patients had to fulfill the criteria for ANCA-associated vasculitis as specified in the 2012 Revised International Chapel Hill Consensus Conference Nomenclature of Vasculitides (17). Only patients with available samples for EM could be included. Samples were either retrieved from tissue obtained by renal biopsy that had previously been stored in glutaraldehyde, or from the paraffin blocks in case of which the quality for EM had to be sufficient for the evaluation of podocyte morphology. Control human renal tissue was used from five pre-transplantation donor kidney biopsies, which showed no abnormalities by LM and from which it was known that the donors were non-proteinuric at time of donation. This study was conducted in accordance with the ethical principles stated in the Declaration of Helsinki.

### Clinical Data

Medical records were used to retrieve data on sex, age, diagnosis (granulomatosis with polyangiitis or microscopic polyangiitis), serology (proteinase 3-[PR3-] or myeloperoxidase-[MPO-]ANCA), and laboratory results (serum creatinine and proteinuria). The estimated glomerular filtration rate (eGFR) at time of biopsy and during follow-up was calculated using the Chronic Kidney Disease Epidemiology Collaboration (CKD-EPI) equation (18). Proteinuria was expressed as total protein excretion in 24-h urine. In case this value was missing, proteinuria by dipstick measurement (scale from negative to +++) was used. All patients were classified as having either moderately or severely increased proteinuria according to the Kidney Disease: Improving Global Outcomes (KDIGO) clinical guidelines: moderately increased proteinuria was defined as a protein excretion rate of 0.15–0.50 g/day, or as trace or + on protein dipstick test; severely increased proteinuria was defined as total protein excretion over 0.50 g/day, or as + or more on protein dipstick (19). Proteinuria levels were assessed at least twice during follow-up: at 10 weeks and 1 year, which were regular moments of outpatient visits for all patients.

### Histopathological Parameters

Renal biopsies were re-evaluated and classified as either focal, crescentic, mixed, or sclerotic class, following the Berden classification (20). Moreover, inflammatory infiltrate (<10%, 10–25%, 26–50%, or >50% of unscarred parenchyma), interstitial infiltrate and tubular atrophy (IFTA [0%, <25%, 26–50%, or >50% of cortical area]), and tubulitis (no mononuclear cells in tubules, foci with 1–4 cells/tubular cross section, foci with 5–10 cells/tubular cross section, or foci with >10 cells/tubular cross section) were determined for each case, according to the Banff classification for allograft pathology (21).

### Measurement of Foot Process Effacement

Renal specimens were fixed in 1.5% GA/1.0% PF fixative or formalin, post-fixed in osmium tetroxide, and embedded in epon (LADD Research Industries Inc., USA). EM sections were stained with uranyl acetate and lead citrate. For each patient and control, 15 pictures were taken with a JEM-1011 electron microscope (JEOL USA, Inc.) at 10.000-fold magnification. As a measure of foot process effacement, FPW was calculated using the formula

$$\frac{\pi }{4}*\frac{\Sigma \text{GBM length}}{\Sigma \text{foot processes}},$$

where ∑foot processes is the total number of foot processes, ∑GBM length is the total length of GBM, and $\frac{\pi }{4}$ is a correction factor for random variation in the angle of section relative to the long axis of the podocyte (9). The total length of GBM in each picture was measured by ImageJ 1.46r software (National Institutes of Health, rsb.info.nih.gov/ij). The number of foot processes was manually counted.

### Measurement of Podocyte Number

We used immunohistochemistry to identify and count podocytes based on staining for WT-1, a podocyte-specific transcription factor (22). Paraffin sections (4-μm thickness) were stained with rabbit anti-human WT-1 (sc-192, Santa Cruz Biotechnology, Dallas, TX, USA), followed by goat anti-rabbit EnVision-HRP conjugate (Dako, Glostrup, Denmark) with diaminobenzidine as the chromogen. The sections were counterstained with hematoxylin. The number of WT-1 positive nuclei per glomerular tuft (referred to as number of podocytes) was counted in three glomeruli unaffected by light microscopic lesions per patient. In the control group, six glomeruli per biopsy were analyzed. The number of podocytes was expressed as number of WT-1 positive nuclei per glomerulus. In the same glomeruli, all nuclei and the surface area of the glomerular tuft were quantified. The software used to count podocytes and nuclei and to measure glomerular surface areas was IMS viewer (Philips Digital Pathology Solution).

### Statistical Analysis

Means were compared between groups by using the student's *t*-test or one-way analysis of variance. Categorical data were compared by using the chi-square test or Fisher's exact test. FPW was correlated to demographic and clinical parameters with Pearson correlation coefficients. All analyses were performed with SPSS statistical software, version 23 (IBM Corp., Armonk, NY, USA). *P* < 0.05 were considered significant.

## Results

### Patient Characteristics

A total of 25 patients were included in this study. The mean ± SD age at biopsy was 55.4 ± 13.5 years, which was similar to the mean age in the control group (47.2 ± 17.3; *P* = 0.24). The 24-hour proteinuria at baseline (proteinuria_0_) was available in 23 patients; the mean was 1.6 ± 1.9 g/day (Table 1). The two patients whose 24-h proteinuria_0_ was unavailable had a positive dipstick (+ and ++ respectively). The mean eGFR at baseline (eGFR_0_) was 42.3 ± 28.6 ml/min/1.73 m^2^. The level of proteinuria_0_ and eGFR_0_ did not correlate (*r* = 0.07; *P* = 0.75), similar to the level of proteinuria_0_ and eGFR at 1 year (eGFR_1year_) (*r* = 0.17; *P* = 0.48). Treatment regimens were as follows: all patients were treated with prednisone; 24 patients received cyclophosphamide, which was switched to maintenance therapy with azathioprine in 17 patients. Six patients received angiotensin converting enzyme—inhibitor (ACE-I) therapy before or after the diagnosis of AAGN; their level of proteinuria_0_ was non-significantly higher than the level in patients who did not receive ACE-I therapy (2.3 ± 2.9 vs. 1.3 ± 1.5 g/day; *P* = 0.45). After 10 weeks of follow-up, the level of proteinuria (proteinuria_10weeks_) was similar in patients receiving ACE-I therapy and patients not receiving ACE-I therapy (1.6 ± 0.9 vs. 1.4 ± 1.6; *P* = 0.76). The levels of proteinuria at 1-year follow-up (proteinuria_1year_) were lower in patients treated with ACE-I compared to patients who did not receive this treatment (0.9 ± 0.8 vs. 0.6 ± 0.9; *P* = 0.58).

**Table 1:** Characteristics of the study cohort and according to FPW.

	**All patients** **(*n* = 25)**	**Patients with FPW <600 nm** **(*n* = 11)^**a**^**	**Patients with FPW ≥600 nm** **(*n* = 10)^**a**^**	***P-*value^**b**^**
Male	15 (60)	6 (55)	6 (60)	1.00
Age, yr	55.4 ± 13.5	51.3 ± 14.4	60.4 ± 13.1	0.15
Diagnosis				0.39
GPA	16 (64)	8 (73)	5 (50)	
MPA	9 (36)	3 (27)	5 (50)	
ANCA serotype				0.43
PR3-ANCA	13 (52)	7 (64)	4 (40)	
MPO-ANCA	9 (36)	4 (36)	4 (40)	
Double positive	2 (8)	0 (0)	1 (10)	
Negative	1 (4)	0 (0)	1 (10)	
Histopathological class				0.03
Focal	13 (54)	9 (82)	3 (33)	
Crescentic/mixed	11 (46)	2 (18)	6 (67)	
Podocytes/glomerulus	15.0 ± 6.5	15.8 ± 6.6	13.4 ± 6.4	0.49
eGFR_0_, mL/min/1.73 m^2^	42.3 ± 28.6	49.4 ± 33.9	38.1 ± 21.4	0.38
eGFR_1year_, mL/min/1.73 m^2^	59.1 ± 23.4	68.4 ± 19.1	57.3 ± 22.5	0.31
Proteinuria_0_, g/day	1.6 ± 1.9	0.9 ± 0.5	2.4 ± 2.7	0.14
Proteinuria_10weeks_, g/day	1.4 ± 1.4	1.0 ± 1.1	2.0 ± 2.0	0.21
Proteinuria_1year_, g/day	0.7 ± 0.9	0.7 ± 1.0	1.0 ± 0.9	0.58
ESRD^c^	3 (12.0)	0 (0.0)	1 (12.5)	0.44

Values are reported as number (%) or mean ± SD.

a FPW could not be measured in four patients, because of insufficient EM material.

b Indicating differences between patients with FPW <600 nm and ≥600 nm.

c *Missing data for two patients due to limited follow-up. eGFR, estimated glomerular filtration rate; ESRD, end-stage renal disease; FPW, foot process width; GPA, granulomatosis with polyangiitis; MPA, microscopic polyangiitis; PR3-ANCA, proteinase 3 ANCA; MPO-ANCA, myeloperoxidase ANCA*.

### Glomerular and Tubulointerstitial Parameters

Thirteen biopsies were scored as focal, five as crescentic, six as mixed, and one could not be classified due to insufficient number of glomeruli (i.e., <7). Patients with a biopsy categorized as focal class had the lowest level of proteinuria_0_ (0.9 ± 0.5 g/day), followed by mixed class (1.2 ± 1.1 g/day), and crescentic class (3.4 ± 3.1 g/day; *P* = 0.02). Proteinuria_10weeks_ did not differ between classes (*P* = 0.39), similar to the level of proteinuria_1year_ (*P* = 0.35). Inflammatory infiltrate, IFTA, and tubulitis were not associated to the level of proteinuria at baseline or during follow-up.

### Foot Process Width

**Figure 1:**
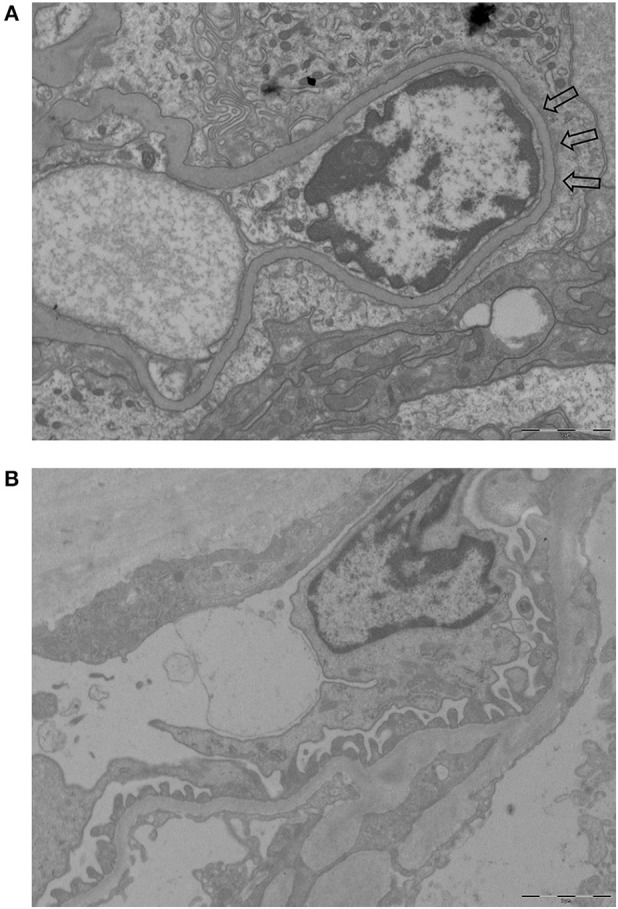
Examples of EM pictures used to calculate FPW (magnification 10.000-fold). **(A)** EM picture of a patient with AAGN showing foot process effacement (part of the effacement is pointed by arrows). **(B)** EM picture of a control with normal foot processes.

**Figure 2:**
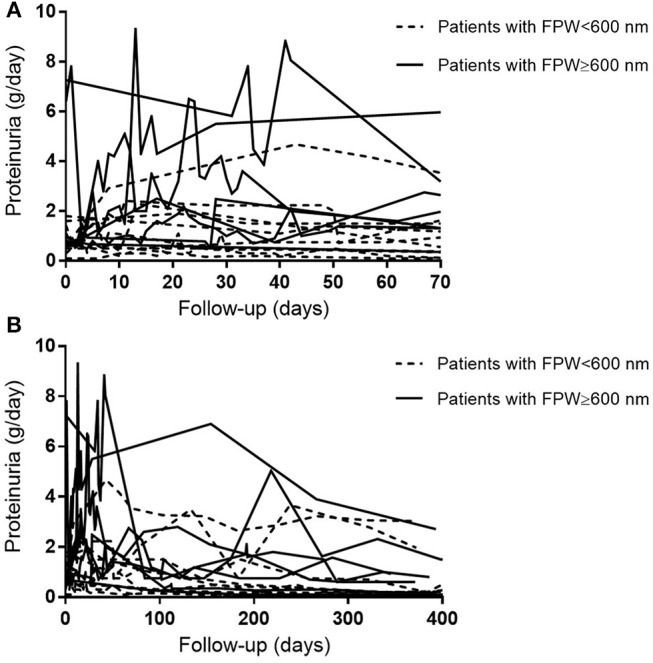
Course of patients' individual 24-h proteinuria levels during follow-up. **(A)** Proteinuria levels during 10 weeks of follow-up. **(B)** Proteinuria levels during 400 days of follow-up.

Figure 1 shows examples of EM pictures from the patient and control group. EM material turned out to be insufficient in four patients. The mean FPW in renal biopsies of 21 patients with AAGN was 603 ± 66 nm. In the control group (biopsies from five living donors), mean FPW was 571 ± 35 nm, which is in accordance with the normal range of FPW as reported in previous studies (7, 9, 10, 16). The mean FPW in patients was not significantly different from the FPW in controls (*P* = 0.31), but the three patients presenting with nephrotic range proteinuria (i.e., >3 g/day) did have a higher FPW compared to controls (657 ± 35 nm; *P* = 0.02). Because the highest FPW in the normal control group was 602 nm, characteristics were compared between patients with FPW <600 and ≥600 nm. Biopsies from patients with a FPW <600 nm were most often categorized as focal class, whereas biopsies with FPW ≥600 nm belonged more often to the crescentic or mixed class (*P* = 0.03; Table 1). Tubulointerstitial parameters were not different between the two groups of FPW. The mean level of proteinuria_0_ was not significantly higher in patients with FPW ≥600 nm compared to patients with FPW <600 nm (2.4 ± 2.7 vs. 0.9 ± 0.5 g/day; *P* = 0.14; Table 1). Figure 2 shows proteinuria levels during follow-up of individual patients according to FPW subgroups. Proteinuria_10weeks_ correlated significantly with FPW (*r* = 0.50; *P* = 0.04). At 1-year follow-up, the correlation between proteinuria and the FPW at biopsy was lost (*r* = 0.22; *P* = 0.40). A correlation of borderline significance was found between FPW and age at biopsy (*r* = 0.43; *P* = 0.05). No significant correlation was observed between FPW and eGFR at baseline and during follow-up.

### Number of Podocytes

**Figure 3:**
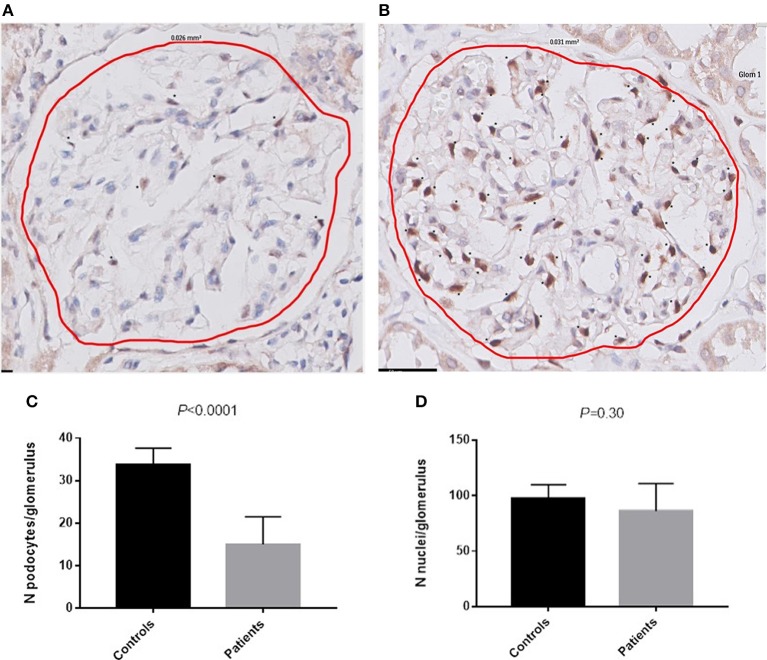
Podocytes positive for WT-1. **(A)** WT-1 staining in a glomerulus of a patient with AAGN. **(B)** WT-1 staining in a glomerulus of a control. Asterisks (*) indicate a podocyte positive for WT-1. **(C)** Number of podocytes per glomerulus in controls and in patients (*P* < 0.0001). **(D)** Number of nuclei per glomerulus in controls and in patients.

Material for immunohistochemistry was available in 19 patients, of which four were excluded due to the absence of at least 3 glomeruli without light microscopic lesions. The remaining 15 patients had a mean of 15 ± 7 podocytes per glomerulus. The mean number of podocytes was 34 ± 4 per glomerulus in the control group, which was significantly higher compared to the patients with AAGN (Figure 3; *P* < 0.0001). The mean surface area of the glomerular tuft was not significantly different in patients vs. controls (0.019 ± 0.006 mm^2^ and 0.025 ± 0.012 mm^2^ respectively; *P* = 0.12); also the total number of nuclei per glomerulus was not significantly different between patients and controls (84 ± 24 and 98 ± 12 respectively; *P* = 0.30). The percentage nuclei positive for WT-1 of the total number of nuclei was significantly lower in patients compared to controls (19.4 ± 9.0% vs. 34.3 ± 1.1%; *P* < 0.001). The number of podocytes per glomerulus in patients with AAGN did not correlate with FPW (*r* = −0.190; *P* = 0.52) or any of the clinical parameters. No significant differences were observed between patients with less and more than the median of 18 podocytes per glomerulus (Table 2).

**Table 2:** Characteristics according to number of podocytes.

	**Patients with <18 podocytes/glomerulus** **(*n* = 9)^**a**^**	**Patients with ≥18 podocytes/glomerulus** **(*n* = 6)^**a**^**	***P*-value**
Male	5 (55.6)	4 (66.7)	1.00
Age, yr	54.2 ± 19.4	58.0 ± 8.4	0.62
Diagnosis			1.00
GPA	5 (55.6)	4 (66.7)	
MPA	4 (44.4)	2 (33.3)	
ANCA serotype			0.61
PR3-ANCA	4 (44.4)	4 (66.7)	
MPO-ANCA	5 (55.6)	2 (33.3)	
Histopathological class			0.59
Focal	3 (37.5)	4 (66.7)	
Crescentic/mixed	5 (62.5)	2 (33.3)	
eGFR_0_, mL/min/1.73 m^2^	34.4 ± 18.7	48.6 ± 14.8	0.14
eGFR_1year_, mL/min/1.73 m^2^	56.3 ± 18.9	59.6 ± 7.2	0.75
Proteinuria_0_, g/day	2.4 ± 2.7	1.6 ± 1.7	0.59
Proteinuria_10weeks_, g/day	1.7 ± 1.9	0.9 ± 0.9	0.46
Proteinuria_1year_, g/day	0.9 ± 0.9	0.3 ± 0.1	0.21
ESRD^b^	0 (0.0)	1 (16.7)	0.46

Values are reported as number (%) or mean ± SD.

a Material for immunohistochemistry was available in 19 patients, of which four were excluded due to the absence of glomeruli without light microscopic lesions.

b *Missing data for two patients due to limited follow-up. eGFR, estimated glomerular filtration rate; ESRD, end-stage renal disease; GPA, granulomatosis with polyangiitis; MPA, microscopic polyangiitis; PR3-ANCA, proteinase 3 ANCA; MPO-ANCA, myeloperoxidase ANCA*.

## Discussion

Previous studies have underlined the importance of proteinuria as a prognostic marker in patients with AAGN (1–4). Since proteinuria has been associated with podocyte abnormalities, we here investigated the structural changes in podocytes in Caucasian patients presenting with AAGN. Although the FPW in patients was not statistically different from the mean FPW in healthy controls, we did identify an interesting association with clinical data as FPW correlated with the level of proteinuria 10 weeks after diagnosis. During these 10 weeks, the level of proteinuria increased in particular in patients whose FPW ≥600 nm (Figure 2B). Therefore, studying podocyte morphology in patients with AAGN may be indicative of whether or not patients will have an increase of proteinuria at short-term follow-up. At 1 year, the correlation between FPW and proteinuria was lost. The anti-inflammatory effect of immunosuppressive therapy may reduce the altered permeability of the glomerular capillary wall, thereby reducing the leak of proteins (23). Moreover, *in vitro* experiments have demonstrated a direct effect of corticosteroids on podocytes, enhancing their survival and promoting their repair (24, 25). Therefore, in addition to reducing inflammation, it could be hypothesized that corticosteroids cause podocytes to regain their normal morphology, leading to the observed decrease in level of proteinuria during 1-year follow-up in our patients with AAGN (Figure 2A). Only by performing EM on repeat protocolized biopsies, which were unavailable in the current study, more insights in this process could be obtained.

The exact relationship between foot process effacement and level of proteinuria is a topic of debate; some studies on glomerular diseases found a correlation between the degree of foot process effacement and amount of proteinuria (6, 7), whereas others did not (9, 10). In our study, FPW did not correlate with the amount of proteinuria at baseline, but we observed severely increased levels of proteinuria at baseline in all patients with a FPW ≥600 nm. Moreover, the three patients presenting with nephrotic range proteinuria had a FPW of 627, 648, and 696 nm; all higher than the highest reported value of 602 nm in controls. These data suggest that foot process effacement and proteinuria are related in patients with AAGN; however, a firm association could not be established.

In contrast to our results, the study by Zou et al. reported a mean FPW of 1269 nm in patients with AAGN, which was significantly higher than the mean FPW of 586 nm they measured in controls (16). In their study, the FPW was higher in patients with elevated serum creatinine (>133 μmol/L). However, they did not find a correlation between FPW and proteinuria at baseline, and did not report on proteinuria during follow-up. Values for FPW in normal controls were similar in the study by Zou et al. and our study, but the mean FPW in our patients with AAGN was much lower than in the Zou study. FPW in our study did correlate to proteinuria levels at 10 weeks. The different results in the study by Zou et al. and ours could have arisen from some differences in study cohorts: 96% of patients from the Zou study were positive for ANCA directed against MPO-ANCA, vs. 36% in our study; and the mean level of proteinuria was higher in their study (2.6 vs. 1.6 g/day in our study). In particular the difference between MPO-ANCA and PR3-ANCA distribution in our study and the study by Zou et al. underlines the differences between Asian and Caucasian patients with AAGN (26, 27). Whether FPW in AAGN varies between populations should be the focus of future studies.

In the current study, we found that biopsies containing a relatively high amount of lesions characteristic for AAGN (i.e., crescentic or mixed class) more often had a FPW ≥600 nm than biopsies with a small number of lesions (i.e., focal class). This is in line with the findings by Zou et al., showing a correlation between FPW and percentage of crescents (16). It has been suggested that podocytes have an active role in crescent formation; in the early stages before crescent formation, they form bridges between the tuft and Bowman's capsule (28). In a later stage, they constitute a component of the crescent, and during the transformation to crescentic cells, they lose podocyte-specific antigens, such as WT-1 (29, 30). In line with this hypothesis, it is telling that we found a 50% decrease in podocytes positive for WT-1 compared to healthy controls, probably reflecting either loss of podocytes or changes in functionality of the podocyte. Our finding of similar numbers of nuclei in glomeruli of patients and controls is suggestive for the latter explanation, and given the diminishment of proteinuria during follow-up this change may be reversible.

The current study has limitations, of which sample size is the major issue. However, EM material of patients with AAGN is scarce, and data on proteinuria are often not routinely documented. We acknowledge that larger studies are required to study podocyte morphology in AAGN into more detail, especially in different populations. Our study included both PR3- and MPO-positive patients, however, perhaps due to limited power, differences in podocyte morphology were not found between different serological phenotypes. Another limitation is that we could not investigate changes in podocyte morphology during follow-up, since repeated biopsy sampling is not part of the standard protocol in AAGN. Moreover, data on factors influencing proteinuria, such as blood pressure, were not available.

In conclusion, we here firstly describe the details of podocyte morphology in Caucasian patients with AAGN. In renal biopsies with AAGN a significant decrease of the podocyte WT-1 marker was found that could be indicative of actual podocyte loss or at least, of a loss of functionality. Patients had variable amounts of FPW, and in particular biopsies with a crescentic or mixed class had the highest FPW. These findings together merit further studies into the morphology and functionality of the podocyte in AAGN. In the meantime, our study indicates that podocyte FPW at baseline could be indicative for proteinuria at short term follow up. Therefore, it would be valuable for prognostic purposes to include a description of the FPW in the diagnostic report of a biopsy with AAGN.

## Data Availability

The raw data supporting the conclusions of this manuscript will be made available by the authors, without undue reservation, to any qualified researcher.

## Ethics Statement

Following the Dutch legislation, this study was not presented to an ethical committee.

## Author Contributions

EvD, AK, and IB designed the study. EvD, LH, AV, and JdZ collected material. EvD and PN performed the FPW measurement with EM and analyzed the results. EvD and MZ performed the WT-1 staining and analyzed these results. All authors contributed to the data interpretation. EvD and IB wrote the manuscript, and all authors reviewed, and approved the manuscript. EvD takes responsibility that this study has been reported honestly, accurately, and transparently, that no important aspects of the study have been omitted and that any discrepancies from the study as planned and registered have been explained.

### Conflict of Interest Statement

The authors declare that the research was conducted in the absence of any commercial or financial relationships that could be construed as a potential conflict of interest.
